# Opioid use and associated factors among pancreatic cancer patients diagnosed between 2007 and 2015

**DOI:** 10.1002/cam4.4610

**Published:** 2022-02-23

**Authors:** Zhanni Lu, Ning Zhang, Sharon H. Giordano, Hui Zhao

**Affiliations:** ^1^ Department of Palliative, Rehabilitation and Integrative Medicine The University of Texas MD Anderson Cancer Center Houston Texas USA; ^2^ Department of Health Services Research The University of Texas MD Anderson Cancer Center Houston Texas USA; ^3^ Department of Breast Medical Oncology The University of Texas MD Anderson Cancer Center Houston Texas USA

**Keywords:** cancer disparities, opioid use, pancreatic cancer, SEER‐Medicare

## Abstract

**Background:**

Opioid therapy provides essential pain relief for cancer patients. We used the population‐based Surveillance Epidemiology and End Results (SEER) linked with Medicare database to identify the patterns of opioid use and associated factors in pancreatic adenocarcinoma cancer patients 66 years or older.

**Patients and Methods:**

We assessed opioid types, dispensed days, opioid uptake rates, and factors associated with opioid use after pancreatic adenocarcinoma cancer diagnosis in Medicare beneficiaries between 2007 and 2015 from the SEER‐Medicare data. Multivariable regression analysis was used to adjust for a variety of patient‐related factors.

**Results:**

We identified a cohort of 10,745 pancreatic cancer patients with a median age of 76 years old and median survival of 7 months; 75% of patients‐initiated opioids after cancer diagnosis. African Americans had the lowest rate of opioid use of 69.1% compared with all other race/ethnicity groups at around 75%. No significant yearly trend of prescribing opioids was detected. Hydrocodone was the most frequently prescribed opioid type. Regression analysis revealed that age ≤80 years, residing in Southern or Western SEER registries, residing in urban/less urban versus big metro areas, having stage IV cancer at diagnosis, longer survival time, and undertaking cancer‐directed treatment or using palliative care were positively associated with opioid initiation, more prescribed opioid types, and higher opioid doses.

**Discussion:**

While a range of sociodemographic variables were associated with opioid use in unadjusted analysis, the associations between race/ethnicity, gender, and socioeconomic status with opioid initiation disappeared when sociodemographic factors, tumor characteristics, and cancer treatment were adjusted.

**Conclusion:**

Health care professionals' opioid prescription pattern for pancreatic cancer patients does not parallel the U.S. opioid epidemic. Racial/ethnic disparities in opioid treatment were not identified.

## Graphical abstract

We determined 75% of pancreatic cancer patients 66 years or older were prescribed opioids between 2007 and 2015. No significant yearly trend of prescribing opioids and hydrocodone was the most frequently prescribed opioid type. Age, sex, geographic location of residence, disease stage, survival time, undertaking cancer‐directed treatment and using palliative care were associated with opioid use among pancreatic cancer patients.

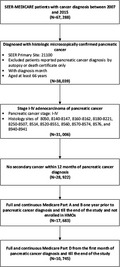

## 1 BACKGROUND

Pancreatic cancer has a high mortality rate, which is largely due to late presentation of symptoms and advanced disease diagnosis. Pain is one of the most common symptoms of pancreatic cancer, affecting more than 80% of pancreatic cancer patients.^1^ As pancreatic cancer progresses, at least 40% of patients experience severe pain, leading to reduced performance status, reduced ability to tolerate cancer treatment and decreased survival.^1^ Opioid‐based pharmacotherapy is the primary strategy to manage moderate and severe pain for patients 65 years or older with progressive and advanced pancreatic cancer in the hope to improve quality of life for these patients.^2^, ^3^ Nearly half of pancreatic cancer patients 65 years or older require use of strong opioids (e.g., morphine) to alleviate severe pain.^4^, ^5^

Pancreatic cancer is the third leading cause of cancer death in the U.S. and more than two‐thirds of pancreatic cancer patients are older than 65 years.^6^, ^7^, ^8^ With the surging aging population in the U.S., incidence of pancreatic cancer is anticipated to rise by 55% by 2030.^6^, ^9^ The increasing incidence of pancreatic cancer in the aging population has created a great need for a better understanding of how these patients use opioids to control pain and symptoms. Most previous studies on patterns of opioid use analyzed patients in different age groups and with different cancer types as a cohort and thus overlooked the heterogeneities of opioid consumption in pancreatic cancer patients, especially among patients 66 years or older.

Thus, we aimed to assess the patterns of opioid use in pancreatic patients older than 65 years diagnosed between 2007 and 2015 using the National Cancer Institute's Surveillance, Epidemiology, and End Results linked with Medicare Database (SEER‐Medicare). Our study focused on pancreatic adenocarcinoma since it is the most common form of pancreatic cancer accounting for more than 90% of pancreatic cancer diagnosis. Using a population‐based approach and a nationwide dataset allowed us to grasp more comprehensively the use of opioids in older pancreatic cancer patients. We also evaluated the association of factors including patients' sociodemographic characteristics and clinical features, including cancer‐directed treatment, with opioid use. Our results could help respond to the soaring urgency of providing effective and safe pain management for older pancreatic cancer patients, in addition to elaborating on racial/ethnic disparities in opioid initiation experienced by this vulnerable population.

## 2 METHODS

### 2.1 Data sources and patients

**FIGURE 1:**
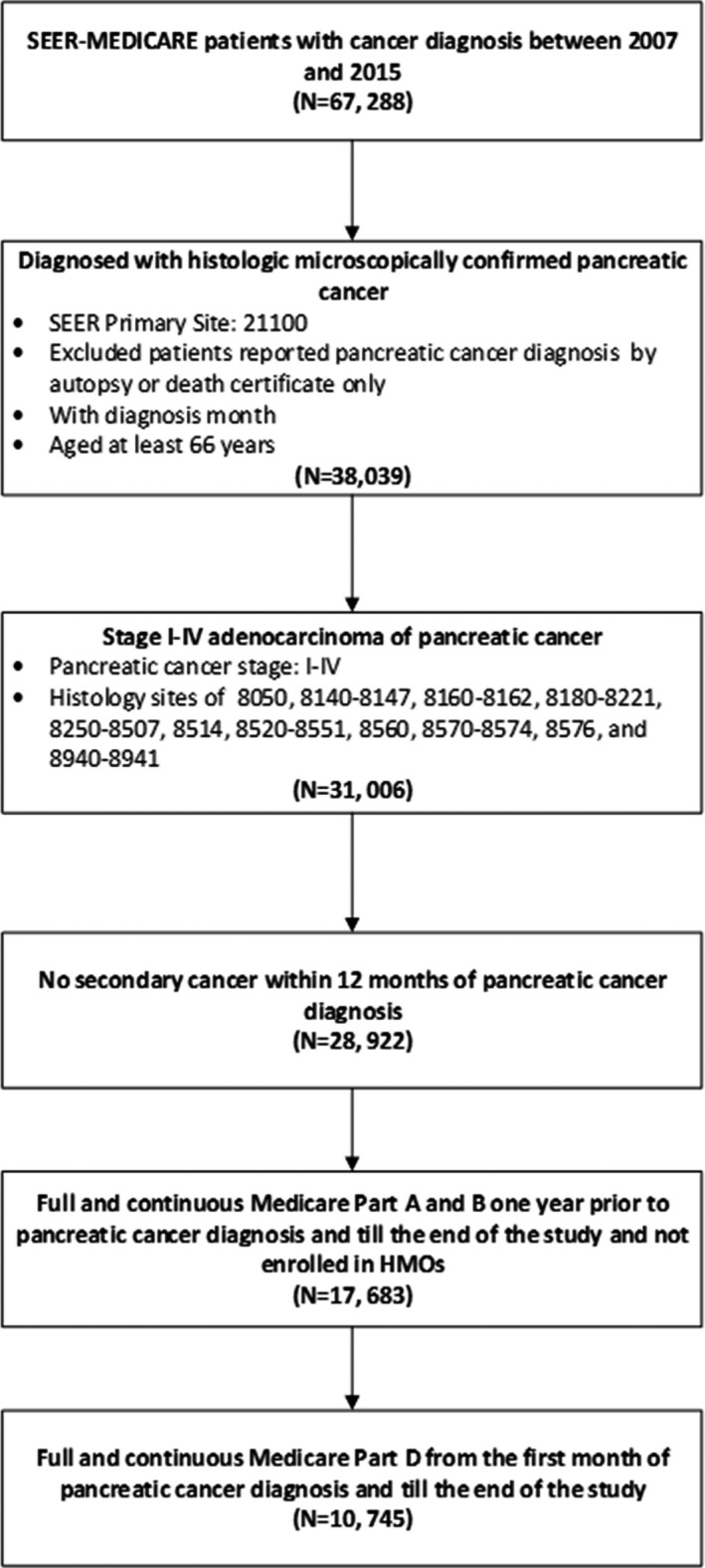
Pancreatic cancer cohort selection consort diagram

The study was exempt by the Institutional Review Board of the University of Texas MD Anderson Cancer Center. Based on the National Cancer Institute's SEER‐Medicare data from 2007 to 2015, we used the following criteria to select the study cohort: (1) being diagnosed with histologically confirmed primary pancreatic cancer between January 1, 2007 and December 31, 2015; (2) the histology type was adenocarcinoma; (3) being at least 66 years old at diagnosis; (4) having full and continuous Medicare Parts A and B coverage from at least 12 months before their pancreatic cancer diagnosis and till the end of the study period, and (5) having full and continuous Medicare Part D from the first month of their pancreatic cancer diagnosis until death or the end of the study. The end of the study period was defined as end of full coverage of Medicare Parts A & B without HMO, end of full coverage of Part D, death, or December 31, 2016 (whichever came first; see Figure 1).

### 2.2 Opioid use and patient covariates

Opioid use was identified from Medicare Inpatient, Outpatient, Hospice, Durable Medical Equipment and Part D files using revenue codes and generic drug names. We examined patients' opioid use by summarizing number of dispense days, opioid dispensed doses, and types of opioid medications in the units of each opioid prescription, each study patient, and the whole study cohort during the study follow‐up duration. Opioid dispensed doses (morphine equivalent doses after pancreatic cancer diagnosis were calculated based on an equation: Strength per Unit multiplied by (Number of Units divided by Days of supply) multiplied by conversion factor.^10^, ^11^ Specifically, we computed, during the study follow‐up duration, (1) the number of patients with at least one opioid prescription; (2) the sum of opioid medication prescriptions a given study patient received; (3) sum of supply days of a given opioid medication and sum of total supply days of all opioid medications prescribed to a given study patient; and (4) sum of dispensed doses of a given opioid medication and sum of total dispensed doses of all opioid medications prescribed to a given study patient, which were reported using median and interquartile measures. Additionally, number of study patients who had their first opioid prescription occurred in the first month after pancreatic cancer diagnosis, and frequencies of opioid medications by opioid types prescribed to the whole study cohort were assessed.

By classifying patients as opioid users versus non‐users, we assessed factors associated with opioid use by using logistic regression analysis. The covariates controlled for in the regression were diagnosis age, diagnosis year, cancer stage, sex, race/ethnicity, marital status, residence urbanization level, SEER registry area, and duration from pancreatic cancer diagnosis to the end of study.^12^, ^13^ Quartile rankings of census‐level median income and the proportion of residents with less than a high school education, and state buy‐in enrollment were used as economic status indicators.^7^, ^8^ The Charlson Comorbidity Index (CCI) was used to classify comorbidity severity based on claims made during the period from 12 months to 1 month before cancer diagnosis.^9^, ^14^ Cancer‐directed surgery, chemotherapy, radiation therapy, immunotherapy were identified using Medicare claims from 1 month before until 6 months after cancer diagnosis.^8^ The International Classification of Diseases, 9th Revision and 10th Revision, Clinical Modification (ICD‐9‐CM and ICD‐10‐CM) codes were used to identify the above cancer treatment and palliative care use (Table A1).^15^, ^16^

### 2.3 Statistical analyses

The Kruskal–Wallis, Mann–Whitney *U*, and chi‐square tests were used to assess the differences of continuous variables and categorical variables by patients' opioid use. Multivariable logistic regression modeling was used to assess factors associated with opioid use and risk ratios (RR) and 95% confidence intervals (95% CI) were reported. The data analysis was conducted using SAS Enterprise Guide 7.1 (SAS Institute, Inc.). All tests were two‐tailed, and the significance level was set at less than 0.05.

## 3 RESULTS

### 3.1 Patient characteristics

We identified 10,745 pancreatic patients, with median age of 76 years old and median survival time of 7 months, of whom 75.0% used opioids. Based on univariable data analysis, patients who were 66–80 years old, women, or married were significantly more likely to use opioids (Table 1). In this initial analysis, African Americans had the lowest opioid use rate of 69.1% compared with any other race/ethnicity in our cohort. Those diagnosed with stage III pancreatic adenocarcinoma had the highest opioid use rate of 79.5% while those with stage IV had the lowest opioid use rate of 73.5%. Further, opioids were more likely to be used in patients who had less comorbidities and survived for 13 months or longer after pancreatic cancer diagnosis. Opioid users were more likely to live in the Southern SEER registries, and in rural areas. Having any cancer‐directed treatment (i.e., radiotherapy, chemotherapy, surgery, or immunotherapy) or using palliative care were also positively associated with opioid use. No significant difference of opioid use was detected by cancer diagnosis year from 2007 to 2015, with the opioid use rate fluctuating between 72.3% and 77.2% over the study period.

**TABLE 1:** Characteristics distribution of pancreatic cancer patients by opioid initiation

Characteristics	Total (*N* = 10,745, Col %)	Used opioids (*N* = 8058, Row %)	No opioid use (*N* = 2687, Row %)	*p* ^a^
Age at diagnosis, *N* (%)				
66–70	2773 (25.81)	2265 (81.7)	508 (18.3)	<0.01
71–75	2799 (26.05)	2243 (80.1)	556 (19.9)	
76–80	1977 (18.4)	1514 (76.6)	463 (23.4)	
≥81	3196 (29.74)	2036 (63.7)	1160 (36.3)	
Age at diagnosis, years, median (IQR)	76 (71, 81)	75 (70, 80)	78 (73, 84)	<0.01
Year of diagnosis, *N* (%)				
2007	908 (8.45)	691 (76.1)	217 (23.9)	0.17
2008	991 (9.22)	733 (74.0)	258 (26.0)	
2009	1033 (9.61)	757 (73.3)	276 (26.7)	
2010	1064 (9.9)	769 (72.3)	295 (27.7)	
2011	1147 (10.67)	873 (76.1)	274 (23.9)	
2012	1193 (11.1)	895 (75.0)	298 (25.0)	
2013	1424 (13.25)	1099 (77.2)	325 (22.8)	
2014	1481 (13.78)	1118 (75.5)	363 (24.5)	
2015	1504 (14)	1123 (74.7)	381 (25.3)	
Sex, *N* (%)				
Male	4611 (42.91)	3412 (74.0)	1199 (26.0)	0.04
Female	6134 (57.09)	4646 (75.7)	1488 (24.3)	
Race/Ethnicity, *N* (%)				
White (non‐Hispanic)	8093 (75.32)	6107 (75.5)	1986 (24.5)	<0.01
African American (non‐Hispanic)	944 (8.79)	652 (69.1)	292 (30.9)	
Asian	740 (6.89)	558 (75.4)	182 (24.6)	
Hispanic	920 (8.56)	704 (76.5)	216 (23.5)	
Marital status, *N* (%)				
Single	982 (9.14)	686 (69.9)	296 (30.1)	<0.01
Married	5544 (51.6)	4310 (77.7)	1234 (22.3)	
Separated/Divorced	1027 (9.56)	791 (77.0)	236 (23.0)	
Widowed	2773 (25.81)	1977 (71.3)	796 (28.7)	
Unmarried/Unknown	419 (3.9)	294 (70.2)	125 (29.8)	
SEER registries^b^, *N* (%)				
East	2515 (23.41)	1781 (70.8)	734 (29.2)	<0.01
Midwest	1343 (12.5)	1000 (74.5)	343 (25.5)	
South	2318 (21.57)	1792 (77.3)	526 (22.7)	
West	4569 (42.52)	3485 (76.3)	1084 (23.7)	
Area of residence, *N* (%)				
Big Metro	6102 (56.79)	4466 (73.2)	1636 (26.8)	<0.01
Metro	3058 (28.46)	2320 (75.9)	738 (24.1)	
Urban/Less Urban	1391 (12.95)	1113 (80.0)	278 (20.0)	
Rural	194 (1.81)	159 (82.0)	35 (18.0)	
Charlson comorbidity score, *N* (%)				
0	4608 (42.89)	3485 (75.6)	1123 (24.4)	<0.01
1	3150 (29.32)	2430 (77.1)	720 (22.9)	
≥2	2987 (27.8)	2143 (71.7)	844 (28.3)	
Poverty level in census tracts, median (IQR)				
1st quartile (least wealthy)	2735 (25.5)	2019 (73.8)	716 (26.2)	<0.01
2nd quartile	2635 (24.5)	2012 (76.4)	623 (23.6)	
3rd quartile	2636 (24.5)	2012 (76.3)	624 (23.7)	
4th quartile (most wealthy)	2739 (25.5)	2015 (73.6)	724 (26.4)	
<12 years of education in census tracts, median (IQR)				
1st quartile (least educated)	2682 (25.0)	2018 (75.2)	664 (24.8)	0.81
2nd quartile	2670 (24.9)	2013 (75.4)	657 (24.6)	
3rd quartile	2701 (25.1)	2011 (74.5)	690 (25.6)	
4th quartile (most educated)	2692 (25.1)	2016 (74.9)	676 (25.1)	
State buy‐in, *N* (%)				
No	10,255 (95.44)	7689 (75.0)	2566 (25.0)	0.87
Yes	490 (4.56)	369 (75.3)	121 (24.7)	
Cancer stage, *N* (%)				
Stage I	927 (8.63)	692 (74.7)	235 (25.4)	<0.01
Stage II	3355 (31.22)	2554 (76.1)	801 (23.9)	
Stage III	1045 (9.73)	831 (79.5)	214 (20.5)	
Stage IV	5418 (50.42)	3981 (73.5)	1437 (26.5)	
Follow‐up months, Median (IQR)	6 (3, 14)	8 (4, 16)	3 (2, 7)	<0.01
Follow‐up months, *N* (%)				
0–2	2260 (21.03)	1107 (49.0)	1153 (51.0)	<0.01
3–5	2610 (24.29)	1948 (74.6)	662 (25.4)	
6–12	2774 (25.82)	2281 (82.2)	493 (17.8)	
≥13	3101 (28.86)	2722 (87.8)	379 (12.2)	
Any cancer treatment, *N* (%)^c^				
None	2000 (18.61)	1174 (58.7)	826 (41.3)	<0.01
Any	8745 (81.39)	6884 (78.7)	1861 (21.3)	
Radiotherapy, *N* (%)				
None	5197 (48.37)	3527 (67.9)	1670 (32.1)	<0.01
Any	5548 (51.63)	4531 (81.7)	1017 (18.3)	
Chemotherapy, *N* (%)				
None	4337 (40.36)	2642 (60.9)	1695 (39.1)	<0.01
Any	6408 (59.64)	5416 (84.5)	992 (15.5)	
Surgery, *N* (%)				
None	8835 (82.22)	6543 (74.1)	2292 (25.9)	<0.01
Any	1910 (17.78)	1515 (79.3)	395 (20.7)	
Immunotherapy, *N* (%)				
None	6249 (58.16)	4569 (73.1)	1680 (26.9)	<0.01
Any	4496 (41.84)	3489 (77.6)	1007 (22.4)	
Palliative care use, *N* (%)				
No	7000 (65.1)	5152 (73.6)	1848 (26.4)	<0.01
Yes	3745 (34.9)	2906 (77.6)	839 (22.4)	
Yes	3745 (34.9)	2906 (77.6)	839 (22.4)	

*Note*: Data are presented as *N* (%) unless otherwise indicated. *p* < 0.05 were considered as statistically significant.

Abbreviations: IQR, interquartile range; SEER, Surveillance, Epidemiology, and End Results.

^a^ *p* values for differences among two groups: chi‐squared and Mann–Whitney *U* test.

^b^ Any cancer‐directed treatment, including any surgery, chemotherapy, radiation therapy, and immunotherapy.

^c^ East (Connecticut and New Jersey), Midwest (Detroit and Iowa), South (Georgia, Kentucky, and Louisiana), West (California, Hawaii, New Mexico, Seattle, and Utah).

### 3.2 Summary of opioid consumption among opioid users

We assessed opioid consumption by the follow‐up time of the study patients during the first 12 months after their pancreatic cancer diagnosis (Table 2). We summarized opioid use in the following four follow‐up intervals: 0–2, 3–5, 6–12, and >12 months. The opioid use rate, number of opioid types prescribed, and total opioid doses consumed significantly increased as the study follow‐up time increased. Patients with follow‐up time of 6–12 months had the highest number of dispense days per opioid type, total opioid dispense days, and doses consumed per opioid type. Hydrocodone was the most frequently prescribed opioid among the study patients followed for at least 2 months or 3 to 5 months. Oxycodone was the most frequently prescribed opioid among those followed for at least 6 months or longer.

**TABLE 2:** Summary of opioid medication use by follow‐up time

Opioid use by follow‐up time	Follow‐up time				
	0–2 months	3–5 months	6–12 months	>12 months	*p* ^a^
Number of patients prescribed at least one opioid, *N* (%)	1107 (49.0)	1948 (74.6)	2281 (82.2)	2722 (87.8)	<0.001
Number of patients initiated opioid in the first month after cancer diagnosis, *N* (%)	967 (42.8)	1254 (48.0)	1160 (41.8)	1033 (33.3)	<0.001
Types of opioid medications prescribed, median (IQR)	2 (1, 3)	3 (2, 5)	5 (2, 9)	5 (2, 13)	<0.001
Dispense days per opioid medication, median (IQR)	14 (8, 20)	15 (9, 21)	15 (9, 21)	13 (8, 20)	<0.001
Dispense days of all opioid medications prescribed, median (IQR)	27 (10, 48)	45 (18,91.5)	70 (22,170)	68 (21,210)	<0.001
Opioid doses* per opioid medication, median (IQR)	45 (30, 75)	48.5 (32.1, 76.3)	50 (33.8, 76.9)	45.6 (32, 69.7)	<0.001
Opioid doses* of all opioid medications prescribed, median (IQR)	86.3 (40, 195.9)	150 (62.1, 346.8)	233.2 (83.9, 620.0)	252.3 (91.1, 727.5)	<0.001
Frequency of types of opioid prescribed in the cohort, *N* (%)					
Fentanyl^b^	299 (12.11)	1031 (13.8)	2089 (13.85)	2667 (9.85)	<0.001
Hydrocodone	773 (31.31)	2298 (30.76)	4186 (27.75)	8141 (30.07)	
Hydromorphone	140 (5.67)	495 (6.63)	1003 (6.65)	1639 (6.05)	
Oxycodone	662 (26.81)	1932 (25.86)	4595 (30.46)	8558 (31.61)	
Morphine	360 (14.58)	963 (12.89)	1697 (11.25)	2413 (8.91)	
Other^c^	235 (9.52)	752 (10.07)	1516 (10.05)	3658 (13.51)	

*Notes:* Opioid doses: The formula to compute opioid doses is (strength per unit)* (daily number of total units)* (morphine milligram equivalent conversion factor).

Abbreviations: Mo: months; IQR: interquartile range.

^a^ *p* values for differences by follow‐up time; Chi‐square/Kruskal–Wallis test.

^b^ One patient could use more than one type of opioid medication.

^c^ Others include buprenorphine, phenylephrine, pentazocine, tramadol codeine, and caffeine.

### 3.3 Factors associated with opioid use

Based on the multivariable logistic regression analysis (Table 3), we found that patients with more than 1 year of follow‐up were 20% more likely to use opioids compared with those with <3 months follow‐up time with 95% CI (1.16, 1.22). Other factors such as being female, residing in the Southern and Western SEER registries, urban/less urban versus big metro environment, surviving longer after pancreatic cancer diagnosis, advanced cancer stage, and undertaking cancer‐directed treatment were associated with higher likelihood of opioid use. Patients older than 80 years were 6% less likely to use opioids compared with those 66–70 years old. After adjusting for patients' sociodemographic characteristics, tumor characteristics, and cancer treatment, African American patients as well as other racial/ethnic minorities had the same odds of receiving opioid medication compared to Whites.

**TABLE 3:** Factors associated with opioid initiation in multivariable logistic regression model

Covariates	Risk ratio (95% CI)	*p*‐Value
Age at diagnosis		
71–75 vs 66–70	1.00 (0.98, 1.02)	0.74
76–80 vs 66–70	0.98 (0.96, 1.00)	0.12
81+ vs 66–70	0.95 (0.93, 0.97)	<0.01
Year of diagnosis		
2008 vs 2007	0.99 (0.96, 1.02)	0.43
2009 vs 2007	0.98 (0.95, 1.01)	0.30
2010 vs 2007	0.98 (0.95, 1.01)	0.19
2011 vs 2007	0.99 (0.96, 1.02)	0.56
2012 vs 2007	0.99 (0.96, 1.02)	0.38
2013 vs 2007	0.99 (0.96, 1.02)	0.55
2014 vs 2007	0.99 (0.96, 1.02)	0.42
2015 vs 2007	0.98 (0.95, 1.01)	0.19
Sex		
Female vs Male	1.01 (1.00, 1.03)	0.07
Race		
African American vs White (non‐Hispanic)	0.99 (0.96, 1.02)	0.44
Asian vs White (non‐Hispanic)	1.00 (0.97, 1.03)	0.88
Hispanic vs White (non‐Hispanic)	1.01 (0.98, 1.04)	0.48
Other/Unknown vs White (non‐Hispanic)	1.01 (0.92, 1.11)	0.82
Martial status		
Single/Never Married vs Married	0.98 (0.96, 1.01)	0.19
Separate/Divorced vs Married	1.00 (0.98, 1.03)	0.78
Widowed vs Married	1.00 (0.98, 1.02)	0.80
Unmarried/Unknown vs Married	0.99 (0.96, 1.03)	0.68
SEER registries		
Midwest vs East	1.01 (0.98, 1.04)	0.46
South vs East	1.03 (1.01, 1.05)	0.01
West vs East	1.02 (1.00, 1.04)	0.04
Urban		
Metro vs Big Metro	1.01 (0.99, 1.02)	0.35
Urban/Less Urban vs Big Metro	1.02 (1.00, 1.05)	0.05
Rural vs Big Metro	1.03 (0.98, 1.09)	0.23
Poverty level in census tracts		
Second quartile vs Lowest quartile	1.01 (0.99, 1.03)	0.53
Third quartile vs Lowest quartile	1.01 (0.98, 1.03)	0.71
Highest quartile vs Lowest quartile	0.99 (0.96, 1.02)	0.60
<12 years of education in census tracts		
Second quartile vs Lowest quartile	1.00 (0.98, 1.02)	0.85
Third quartile vs Lowest quartile	1.00 (0.98, 1.02)	0.93
Highest quartile vs Lowest quartile	1.01 (0.98, 1.03)	0.60
Charlson comorbidity		
1 vs 0	1.01 (1.00, 1.03)	0.13
2+ vs 0	1.01 (0.99, 1.03)	0.23
Follow‐up months		
3–5 vs 0–2	1.12 (1.10, 1.15)	<0.01
6–12 vs 0–2	1.16 (1.13, 1.19)	<0.01
≥13 vs 0–2	1.19 (1.16, 1.22)	<0.01
State buy‐in		
Yes vs No	1.03 (0.99, 1.07)	0.16
Cancer stage		
Stage II vs stage I	1.00 (0.97, 1.02)	0.84
Stage III vs stage I	1.02 (0.99, 1.05)	0.22
Stage IV vs stage I	1.04 (1.01, 1.06)	0.01
Cancer treatment		
Yes vs No	1.03 (1.01, 1.05)	0.01
Palliative care		
Yes vs No	1.01 (0.99, 1.02)	0.21

Abbreviation: CI, confidence interval.

SEER registries: East (Connecticut and New Jersey), Midwest (Detroit and Iowa), South (Georgia, Kentucky, and Louisiana), West (California, Hawaii, New Mexico, Seattle, and Utah).

Cancer treatment: Any surgery, chemotherapy, radiation therapy, and immunotherapy.

**TABLE A1:** Medicare claims codes to define pancreatic cancer treatments and palliative care

Variables	ICD‐9 diagnosis codes	ICD‐10 diagnosis codes	ICD‐9 procedure codes	ICD‐10 procedure codes	CPT/HCPCS Codes	Revenue codes
Chemotherapy	99.25	3E03305	V58.11, V66.2, V67.2	Z5111, Z5189, Z08, Z09	96,400–96,549, J8520, J8521, J8530, J8540, J8560, J8597, J8610, J8999, J9000‐J9999 (except J9003, J9165, J9175, J9202, J9209, J9212‐J9226, J9240, J9395), Q0083‐Q0085	0331,0332, 0335
Radiation treatment	92.2, 92.20‐92.27, 92.29, 92.3, 92.30‐92.39, 92.4, 92.41	3E0J304, 3E0J704, 3E0J804, DFY37ZZ, DF030ZZ, DF1397Z, DF1398Z, DF1399Z, DF139BZ, DF139CZ, DF139YZ, DF13B7Z, DF13B8Z, DF13B9Z, DF13BBZ, DF13BCZ, DF13BYZ, DF030ZZ, DF031ZZ, DF032ZZ, DF033ZZ, DF034ZZ, DF035ZZ, 0FHD01Z, 0FHD31Z, 0FHD41Z, 0FHD71Z, 0FHD81Z, DFY3CZZ, DFY3FZZ, D020DZZ, D021DZZ, D027DZZ, DG20DZZ, D020DZZ, D021DZZ, D027DZZ, DG20DZZ, D020JZZ, D021JZZ, D027JZZ, DG20JZZ, D020HZZ, D021HZZ, D027HZZ, DG20HZZ, D020DZZ, D021DZZ, D027DZZ, DG20DZZ, DF033Z0	V58.0, V66.1, V67.1	Z510, Z5189, Z08, Z09	77, 371–77, 373, 77, 401‐77, 525, 77, 761–77, 799, G0174, G0251, G0339, G0340	0330, 0333
Surgery	52.51–52.53, 52.59, 52.6, 52.7	0FBG0ZZ, 0FBG3ZZ, 0FBG4ZZ, 0FBG8ZZ, 0DB90ZZ, 0DB93ZZ, 0DB94ZZ, 0DB98ZZ, 0FBG3ZZ, 0DB90ZZ, 0FBG3ZZ, 0DB93ZZ,0FBG3ZZ, 0DB94ZZ, 0DB97ZZ, 0FBG0ZZ, 0FBG3ZZ, 0FBG4ZZ, 0FBG8ZZ, 0FTG0ZZ, 0DT90ZZ, 0FTG0ZZ, 0FTG4ZZ, 0FTG0ZZ, 0DT90ZZ, 0FTG0ZZ, 0DT94ZZ, 0FTG0ZZ, 0DT97ZZ, 0FTG0ZZ, 0DT98ZZ, 0FTG4ZZ, 0DT90ZZ, 0FTG4ZZ, 0DT94ZZ, 0FTG4ZZ, 0DT97ZZ, 0FTG4ZZ, 0DT98ZZ, 0FTG0ZZ, 0DT90ZZ, 0F190Z3, 0F1G0ZC, 0D1607A, 0D160JA, 0D160KA, 0D160ZA	N/A	N/A	48,120,48,145,48,146,48,150,48,152‐48,155,48,160	N/A
Immunotherapy	N/A	N/A	V58.12	Z5112	N/A	N/A
Palliative Care	V66.0‐ V66.9	Z51.5	N/A	N/A	N/A	N/A

## 4 DISCUSSION

Safe, effective, and successful opioid‐based pain management is an integral component of health care for pancreatic cancer patients, which is considered an indicator of high‐quality end‐of‐life care.^17^, ^18^ We determined 75% of pancreatic cancer patients 66 years or older were prescribed opioids during the study period. No significant yearly trend of prescribing opioids to pancreatic cancer patients 66 years or older was identified. Hydrocodone was the most frequently prescribed opioid type. Age, sex, geographic location of residence, disease stage, survival time, undertaking cancer‐directed treatment, and using palliative care were associated with opioid use among pancreatic cancer patients.

Consistent with our finding that younger patients, being female, undergoing cancer‐directed treatment, and having longer survival time were more likely to use opioids, Fisher et al. reported that opioids were less likely to be prescribed for those who were older, male, and with less than 6 months of life expectancy. Older cancer patients are less likely to be prescribed opioids because various factors can affect their opioid responses, including comorbidities, metabolism, and genetic factors, according to previous studies.^19^, ^20^, ^21^ Palliative care use was associated with more frequent prescription of opioids in our univariable analysis. Wang et al.,^22^ Tse et al.,^23^ and Fisher et al.,^24^ reported older pancreatic cancer patients using palliative care consumed opioids significantly more frequently than those receiving standard oncology care. We suggest that the higher likelihood of using opioids among those with palliative care use might be attributed to the practice of prescribing strong and high dose opioids to manage severe pain for cancer patients in the palliative care stage.^17^, ^18^

Our data also indicated that patients with longer follow‐up time, less comorbidities, and continuing with cancer‐directed treatment consumed more types and higher doses of opioids, which might be due to opioids being the first line pharmacotherapy for controlling pain or symptoms resulting from undertaking cancer‐directed treatment for a longer time. Mercadante et al.^25^ demonstrated cancer patients' consumption of opioid doses increased as their illness progressed. Our study found that patients residing in the Southern and Western SEER Registries, semi‐rural areas were more likely to use opioids. Such geographic variations in opioid use in the U.S. were reported in Beccaro et al.,^26^ Stiefel et al.,^27^ and Roeland et al.^28^ We argue that pancreatic adenocarcinoma cancer patients' geographic location was one of the main factors influencing the access to and consumption of opioids.^29^, ^30^

In our study, we found no racial/ethnic disparities in opioid use among pancreatic cancer patients after adjusting for patients' sociodemographics, tumor characteristics, and cancer treatment. Although African Americans had the lowest opioid initiation rate in our univariable analysis, this difference disappeared after adjusting for the aforementioned covariates in multivariable logistic regression analysis. In contrast to our finding, a previous study by Pletcher et al.^31^ reported Black patients were less likely to receive opioid analgesic medication than White patients to manage their pain, with odds ratio of 0.66 and 95% CI (0.62, 0.70). Several factors may explain the discrepancy between our findings and those of Pletcher and colleagues. Pletcher's study was based on data collected from emergency department visits, and the study population was not cancer patients. Moreover, the data had limited information on patients' chronic disease conditions and disease‐related treatment.^31^ In our study, patients' opioid use was obtained from the full spectrum of care including inpatient and outpatient services. Moreover, we found that opioid use was significantly associated with a range of variables including patients' age, cancer stage, cancer treatment, and follow‐up time. Therefore, racial/ethnic disparities of opioid use were no longer significant after adjusting for patients' sociodemographics, tumor characteristics, cancer treatment, follow‐up time, and comorbidity in the multivariable model.

Our study has many strengths. We used the population‐based and longitudinal SEER‐Medicare data to identify the patterns and factors associated with opioid use in pancreatic cancer patients 66 years or older. Previous studies of the patterns of opioid use had limited sample sizes or focused on multiple cancer types, making it a challenge to generalize the study results to pancreatic cancer patients. Our results, generated from a large sample size of older pancreatic cancer patients, present a more accurate assessment of changing patterns of opioid use along the disease trajectory for this population. Further, our analysis of opioid use stratified by the study follow‐up time revealed the complex interactions between opioid use and pancreatic cancer survival time.

Despite these strengths, our study had some limitations. Since we used administrative data, no information regarding patients' preference of pain medications, oncologists' training in opioid prescribing, and regulatory policies surrounding opioid prescribing was captured, which might have led to selection bias and uncontrolled confounders. As our study results were based on a cohort of Medicare beneficiaries with median age of 76 years old, the generalizability of the results to younger pancreatic cancer patients remains unknown. Further, due to using the SEER‐Medicare data from 2007 to 2015 to assess the utilization patterns of opioids among pancreatic cancer patients, the follow‐up time of our study patients was limited by the availability of our study data and the unique characteristics of claims data. We might overlook the information on study patients' opioid use if their opioid use was not covered by their Medicare health insurance coverage periods available in our study data or the study patients remained alive after December 31, 2016. Yet, 95.1% of total 10,745 patients ended their follow‐up time due to death. Thus, our study fully captured majority of our study cohort's use of opioids from pancreatic diagnosis to their death. The likelihoods of using opioids in the study patients died during the study follow‐up period increased significantly along with the increasing of patients' survival time. Also, since our study time ended in 2015, it will be interesting to determine if our conclusions remain unchanged for more recent years by analyzing newer SEER‐Medicare data.

Our study indicated that the study patients with longer survival time were more likely to use opioids and used more different types of opioids. Yet, those survived more than 12 months were less likely to initiate opioid use during the first month after cancer diagnosis than those survived less. Such findings indicated potential relations between opioid initial prescription timing and survival time in pancreatic cancer patients, echoing the argument of the correlations between opioid initial prescription timing and prolonged opioid use in post‐surgery patients.^32^ Clinicians need to take into consideration the timing of starting prescribing opioids for opioid naïve patients 65 or older with pancreatic cancer to ensure the optimized pain management and avoidance of possible opioid overuse. Additionally, our study found that fentanyl was more frequently used in those survived 4–12 months. Oxycodone was most used in those survived for more than 12 months. Fentanyl and oxycodone have been associated with overdose deaths from synthetic opioids according to CDC in the United States.^33^ Although pancreatic cancer patients 65 years or older are not a high‐risk population of overdose deaths from fentanyl and oxycodone, clinicians need to consider the effects of prolonged use of fentanyl and oxycodone in this population given their unpredictable responses to opioids.

Using opioid‐based pharmacotherapy to manage cancer pain remains an essential component to improve pancreatic adenocarcinoma cancer patients' quality of life. A consensus understanding of opioid use is that opioids can control cancer pain effectively when prescribed and used correctly and safely.^19^ Our results could help health care professionals make clinical decisions on balancing opioid‐based pain management and safe opioid prescribing for pancreatic adenocarcinoma cancer patients 66 years or older while facing their unpredictable opioid responses, demand for high quality of life, and the ongoing crisis of opioid misuse and overuse in the U.S.

## ETHICAL APPROVAL

This study was considered exempt of institutional review board approval because the data are de‐identified.

## CONFLICT OF INTEREST

No conflict of interest to disclose.

## AUTHOR CONTRIBUTIONS

Zhanni Lu: Investigation, data analysis and interpretation, methodology, visualization, writing–original draft, writing review, and editing. Ning Zhang: Investigation, data analysis and interpretation, methodology, visualization, writing–original draft, writing review, and editing. Hui Zhao: Investigation, analysis and interpretation, methodology, software, writing review and editing, and supervision. Sharon H. Giordano: Investigation, data acquisition, interpretation, editing, and supervision.

## FUNDING INFORMATION

This study was partially supported by the National Institutes of Health through a Cancer Center Support grant (P30 CA016672), and by the Duncan Family Institute. Dr. Giordano is supported by a Cancer Prevention & Research Institute of Texas grant (RP160674), and Komen SAC150061.

## Data Availability

The datasets generated for this study are based on SEER‐Medicare data with cancer cases diagnosed between 2007‐2015 and claims till December 2016. The data can be purchased through the National Cancer Institute: https://healthcaredelivery.cancer.gov/seermedicare/obtain/cost.html.
